# Comparison of Three In-House Real PCR Assays Targeting Kinetoplast DNA, the Small Subunit Ribosomal RNA Gene and the Glucose-6-Phosphate Isomerase Gene for the Detection of *Leishmania* spp. in Human Serum

**DOI:** 10.3390/pathogens10070826

**Published:** 2021-06-30

**Authors:** Konstantin Tanida, Carsten Balczun, Andreas Hahn, Alexandra Veit, Beatrice Nickel, Sven Poppert, Patrick Leander Scheid, Ralf Matthias Hagen, Hagen Frickmann, Ulrike Loderstädt, Egbert Tannich

**Affiliations:** 1 Department of Microbiology and Hospital Hygiene, Bundeswehr Hospital Hamburg, 20359 Hamburg, Germany; konstantin.tanida@gmail.com; 2 Department XXI, Microbiology and Hospital Hygiene, Section B, Bundeswehr Central Hospital Koblenz, 56070 Koblenz, Germany; carstenbalczun@bundeswehr.org (C.B.); patrickscheid@bundeswehr.org (P.L.S.); ralfmatthiashagen@bundeswehr.org (R.M.H.); 3 Department of Medical Microbiology, Virology and Hygiene, University Medicine Rostock, 18057 Rostock, Germany; hahn.andreas@me.com; 4 Bernhard Nocht Institute for Tropical Medicine Hamburg, National Reference Centre for Tropical Pathogens, 20359 Hamburg, Germany; veit@bnitm.de (A.V.); tannich@bnitm.de (E.T.); 5 Swiss Tropical and Public Health Institute, 4051 Basel, Switzerland; beatrice.nickel@swisstph.ch (B.N.); sven@poppert.eu (S.P.); 6 University of Basel, 4001 Basel, Switzerland; 7 Department of Hospital Hygiene & Infectious Diseases, University Medicine Göttingen, 37075 Göttingen, Germany; ulrike.loderstaedt1@med.uni-goettingen.de

**Keywords:** leishmania, visceral, Kala Azar, real-time PCR, test comparison, in-house, serum

## Abstract

To perform PCR from serum for the diagnosis of visceral leishmaniasis is convenient and much less invasive than the examination of deeper compartments such as bone marrow. We compared three *Leishmania*-specific real-time PCRs with three different molecular targets (kinetoplast DNA, the small subunit-ribosomal RNA-(ssrRNA-)gene, the glucose-6-phosphate isomerase-(*gpi*-)gene) regarding their sensitivity and specificity in human serum. Residual sera from previous diagnostic assessments at the German National Reference Center for Tropical Pathogens Bernhard Nocht Institute for Tropical Medicine Hamburg and the Swiss Tropical and Public Health Institute were used. The sensitivities of kinetoplast DNA-PCR, ssrRNA-gene PCR, and *gpi*-PCR were 93.3%, 73.3%, and 33.3%, respectively, with 15 initial serum samples from visceral leishmaniasis patients, as well as 9.1%, 9.1%, and 0.0%, respectively, with 11 follow-up serum samples taken at various time points following anti-leishmanial therapy. Specificity was 100.0% in all assays as recorded with 1.137 serum samples from deployed soldiers and migrants without clinical suspicion of visceral leishmaniasis. Kinetoplast-DNA PCR from serum was confirmed as a sensitive and specific approach for the diagnosis of visceral leishmaniasis. The results also indicate the suitability of serum PCR for diagnostic follow-up after therapy, in particular regarding therapeutic failure in case of persisting positive PCR results.

## 1. Introduction

As reviewed recently [1], the diagnosis of causative agents of visceral leishmaniasis is mainly based on microscopical, serological, and nucleic acid amplification testing (NAT) procedures. NAT-based approaches are known to be highly sensitive and are thus recommended for oligoparasitic leishmaniasis such as leishmaniasis recidivans or mucosal leishmaniasis [2]. For a disease with high parasitemia, such as symptomatic visceral leishmaniasis, it is even discussed that NAT might be too sensitive, so it will detect asymptomatic, self-limiting infections without clinical implications as well [3]. As known from study results from Brazil, ratios of asymptomatic infections to clinically apparent visceral leishmaniasis may range from 6.5:1 up to 18:1 [4,5,6], so infection events do not necessarily require medical treatment. On the contrary, even in the case of clinically relevant disease, the persistence of parasites in well-vascularized lymphatic nodes in spite of successfully cured infections will be no exemption [7], also limiting the value of real-time PCR for therapy control.

However, the persistence of viable parasites in patients after clinically successful therapy may lead to reactivation. Further, opportunistic infections in immunocompromised individuals, e.g., due to acquired immunosuppression syndrome (AIDS), cancer, malnutrition, or immunosuppressive therapy, are associated with an increased risk of progressive disease [8]. Therefore, even asymptomatic infections have been shifted into the focus of medicine because, initially, asymptomatic infection might progress to clinical disease in the event of later immunosuppression. Indeed, scientists within the US armed forces medical service were able to demonstrate such asymptomatic infections in deployed soldiers in a recently published assessment [9].

In this American study [9], however, only a minority of patients with immunologically relevant contact to *Leishmania* spp., as indicated by serology, were also positive by PCR. Of note, serology cannot discriminate active from previous, already cured infections, while the sensitivity of PCR depends on the sample material assessed with invasively taken materials such as bone marrow and less invasively acquired samples, such as Buffy coat, whole blood, and serum in declining order of sensitivity. Furthermore, a relatively low degree of standardization has been described for molecular in-house diagnostic approaches for leishmaniasis [10]. Quantitative PCR has been suggested as an option for the discrimination between asymptomatic infections and visceral disease [11,12]. Commercial diagnostic solutions are still scarce.

In spite of the poor standardization, very sensitive screening PCRs for leishmaniasis have been described, which are able to detect *Leishmania* spp. DNA even from fixed and stained slides [13]. Additionally, multinational test comparisons of screening PCRs targeting *Leishmania* spp. DNA have been performed [14], confirming the very high sensitivity of these real-time PCR approaches.

In the study described here, we compared the diagnostic performance of three in-house real-time PCRs for *Leishmania* spp. DNA, comprising the protocols targeting kinetoplast DNA [15], the small subunit ribosomal RNA-(ssrRNA-)gene [16], and the glucose-6-phosphate isomerase-(*gpi*-)gene, respectively. The latter assay was based upon newly designed oligonucleotides, but the target gene is known from previously described diagnostic PCR protocols [17,18] and even from molecular typing schemes for *Leishmania* spp. [19]. The kinetoplast DNA PCR protocol [15], of note, has recently also been successfully evaluated in the course of a PCR test comparison, including samples from patients with cutaneous leishmaniasis [20]. For the test comparison described here, residual sera and follow-up sera from visceral leishmaniasis patients were used as positive controls. As presumptive but only inconsistently serologically confirmed negative controls, residual sera from German soldiers returning from mission areas as well as residual sera from migrants without clinical hints for visceral leishmaniasis were used for specificity assessment only. They were not included in sensitivity assessment, keeping in mind that asymptomatic infections with parasites causing visceral leishmaniasis have been described among returning military personnel [9] as well as among inhabitants of endemic areas [21].

## 2. Results

A total of 26 serum samples from 18 patients with clinical and diagnostically confirmed visceral leishmaniasis (see: Methods) were assessed by real-time PCR targeting parasite kinetoplast DNA, the small-subunit-ribosomal RNA (ssrRNA) gene as well as the *gpi* gene. The samples comprised 15 initial samples and 11 follow-up samples as received by the involved diagnostic institutions. Corresponding clinical data (such as status before or after therapy) were not consistently available. Furthermore, there were no data on whether the diagnosing institutions (Bernhard Nocht Institute for Tropical Medicine Hamburg and Swiss Tropical and Public Health Institute) were really the first institutions to set up the diagnosis or just contacted for the purpose of confirmatory testing. Residual material from initial samples was not available anymore for all follow-up samples. The periods between initial and follow-up sampling ranged from few days to 22 months (Table 1).

Regarding the 15 initial samples, the kinetoplast DNA-PCR was positive in 14/15 cases (93,3%), the ssrRNA-gene-PCR in 11/15 cases (73.3%), and the *gpi*-PCR in 5/15 cases (33.3%). In the case of the follow-up samples, both the kinetoplast DNA-PCR and the ssrRNA-gene-PCR showed 1 positive sample among the 11 samples assessed (9.1%), while the *gpi*-PCR remained negative in all 11 instances (0.0%). Regarding the recorded cycle threshold (Ct) values, median Ct value (minimum, maximum) showed a significant difference in Kruskal Wallis testing (*p* = 0.0038) between the assays with 23 (10, 39) for the kinetoplast DNA-PCR, 27 (17, 32) for the ssrRNA-gene PCR, and 38 (36, 39) for the *gpi*-PCR, respectively. Thereby, Dunn’s multiple comparisons tests indicated statistically significant differences between the *gpi*-PCR and both the kinetoplast DNA PCR (*p* < 0.01) and the ssrRNA-gene-PCR (*p* < 0.05), while no significance was detectable for differences of the Ct-values when comparing the latter two PCRs.

In a total of 1137 serum samples from migrants and deployed soldiers without confirmed but also without consistently excluded visceral leishmaniasis as detailed in the methods chapter, none of the assessed real-time PCRs showed a positive signal for *Leishmania* spp., suggesting reliable specificity of all assays. Only the *gpi*-PCR showed a non-interpretable late signal (Ct value >40) in a serum sample from a migrant from Afghanistan. Those results suggest excellent specificity of virtually 100.0% in all assays in case of an application with serum obtained from returnees and migrants from the subtropics and tropics, while lacking reference testing did not allow sensitivity estimations with these samples.

## 3. Discussion

The study was performed to comparably assess sensitivities and specificities of three in-house real-time PCR assays with different target sequences for the diagnosis of visceral leishmaniasis in serum samples. The examination of the initial samples from visceral leishmaniasis patients resulted in nearly 100% sensitivity for a real-time PCR targeting kinetoplast DNA. Only one out of fifteen initial serum samples of visceral leishmaniasis patients was not positively detected. In this sample, however, PCR from the bone marrow had also indicated a very late Ct value. Accordingly, it is likely that this presumed “initial” sample might, in fact, have been a sample collected for confirmatory purposes after the initiation of the therapy. This could explain both the low copy number in the bone marrow and the failed diagnosis in the serum sample. In contrast, the ssrRNA-gene PCR results indicated slightly lower sensitivity, while the *gpi*-PCR showed insufficient sensitivity when used for the screening of serum samples. The latter result was underlined by considerably higher Ct values in *gpi*-PCR compared to both kinetoplast DNA PCR and ssrRNA-gene PCR. Of note, the ssrRNA-gene PCR comprised only 40 cycles in line with the published protocol [16] and thus five cycles less than the two competitor protocols. The practical relevance of this hypothetical bias for the assay comparison is, however, questionable, as Ct values >32 were never observed with clinical serum samples applying this assay.

The lower sensitivity of the *gpi*-PCR is in line with a recorded higher limit-of-detection (see: methods) even with reference DNA material. So, the oligonucleotide binding of the kinetoplast DNA-PCR and the ssrRNA-gene PCR was presumably better. Further, the diagnostic sensitivity with the clinical samples is likely to have been affected by multiple repeats of the ssrRNA-gene and the targeted kinetoplast DNA-sequence within the parasites [22,23].

In follow-up samples from patients with anti-leishmanial therapy, PCR results in serum turned out to be negative. In 1 out of 11 follow-up samples only, the kinetoplast DNA-PCR and the ssrRNA-PCR showed a positive signal, respectively. This single positive follow-up sample, however, had been received just a few days after the initial sample for confirmatory testing. In contrast, negative PCR results in all assessed assays were recorded for all follow-up samples with time periods of several months after the initiation of therapy. Although the available clinical data were insufficient to definitely prove that the absence of detectable *Leishmania* spp. DNA from follow-up serum samples was associated with anti-leishmanial therapy, and it seems obvious to assume that the presence of persisting positive serum PCR after some months may confirm a therapeutical failure.

The use of kinetoplast DNA-PCR from human serum for the diagnosis of visceral leishmaniasis has been suggested previously [24,25] but is currently not included, e.g., in the German guidelines for the diagnosis and treatment of visceral leishmaniasis [26]. In a previous study including Mediterranean patients with visceral leishmaniasis, kinetoplast DNA-PCR from serum showed a sensitivity of 97% and a specificity of 95%, respectively, when used for primary diagnosis of visceral leishmaniasis [24]. Accordingly, this recent study suggested similar reliability as our assessment. In a study from Brazil, in contrast, an excellent specificity of 100% but reduced sensitivity of only 85% for kinetoplast DNA-PCR from serum compared to 97% sensitivity from whole blood has been reported [25]. It remains uncertain whether this reduced sensitivity resulted either from the genetic variation of South American *Leishmania* spp. causing visceral leishmaniasis or from discrepant nucleic acid extraction strategies. In the study presented here, different nucleic acid extraction methods could not be compared due to insufficient residual samples volumes. So, the standardized automated approach as described in the methods chapter was chosen. The EZ1 Virus Mini Kit, in particular, was chosen because it is well suited for the extraction of free nucleic acid sequences from various body fluids in line with our experience from the diagnostic routine. The residual sample volumes available for this study were, unfortunately, insufficient to comparably assess different nuclei acid extraction protocols.

Regarding the assays’ specificity, none of the assessed real-time PCRs showed false-positive signals in the samples from returned soldiers [27] or migrants [28], which were solely used for specificity assessment because asymptomatic infections with *Leishmania* spp. were not definitely excluded. Accordingly, these samples can be considered as presumptive negative controls only. Nevertheless, the results suggested a specificity of virtually 100% for all tested PCR assays for the diagnosis of *Leishmania* spp. in human serum. Just one very late borderline signal, which was not to be interpreted as positive, was observed in the *gpi*-PCR with a sample of a migrant from Afghanistan, a region where visceral leishmaniasis is extremely infrequent or even absent [29]. Clinical signs of cutaneous leishmaniasis were not detected in this patient as well [28]. It remains unproven whether comparable specificity results would have been achieved if samples from areas of endemicity for phylogenetically closely related pathogens such as *Trypanosoma* spp. were included, although Southern American evaluation data indicate acceptable specificity, too [30]. The specificity control population used was more representative for a travel clinic in a non-endemic area. However, at least cross-reactions with reference DNA of relevant protozoan parasites potentially occurring in human blood samples were not detected in the real-time PCR assays assessed.

As an interesting side effect, we did not find any hint of the presence of visceral leishmaniasis in the samples of the German deployed soldier subpopulation, which is congruent to the findings in a previous clinical study [27]. Asymptomatic carriage of *Leishmania* spp. DNA, as reported for US soldiers [9] and even for soldiers from neighboring Austria [31] after deployment as well as for individuals in endemic areas [21], was not confirmed in the present study as a frequent phenomenon for the sampled subpopulations of German soldiers showing up for post-deployment assessments after tropical deployments [27] and of migrants from areas of endemicity [28].

Of note, diagnostic *Leishmania*-PCR from urine samples [32,33,34,35] as well as various associated nucleic acid extraction strategies [36] have been introduced and recently also summarized in reviews [37,38]. Published interpretations regarding the diagnostic reliability of these approaches vary [37,38]. From the patients assessed in this study, no residual urine samples were available, so any conclusions on the diagnostic accuracy of urine-based PCR-diagnosis of visceral leishmaniasis are beyond the scope of this work.

Admittedly, the study has a number of limitations. First, in spite of the multi-centric cooperation, the scarce availability of positive sample material from visceral leishmaniasis patients limited the statistical value of this assessment. Therefore, the present study was only performed “descriptively” without any further sophisticated statistical approaches. Second, both the demands by the ethical clearance for test comparison purposes and the partly scarce availability of such information in the laboratory information systems have prevented the inclusion of patient-specific data, an admitted violation of the STARD (Standards for Reporting Diagnostic Accuracy) criteria [39]. Third, the lack of information on the applied anti-leishmanial therapy and the lack of standardization of follow-up sampling hampered a more detailed examination of serum PCR for therapy control, a consequence of the retrospective design of this evaluation study. Fourth, the lack of previous systematic assessment of the negative control population regarding asymptomatic visceral leishmaniases allowed merely a specificity analysis with these samples. In contrast, a qualified statement on the assays’ sensitivity in the case of potential asymptomatic persistence of *Leishmania* spp. in the patients’ bone marrow could not be given. Unfortunately, serological assessment of the presumptive negative control samples would have been beyond the financial limits of this study, and samples from patients with known asymptomatic infections were not available for inclusion. Fifth, DNA degradation might have occurred over the years within the serum samples in spite of optimized storage conditions at -80°C. Interestingly, however, leishmanial DNA in serum was still detectable even after a maximum of 26 years in the used reference materials. Hypothetically, such positive PCR signals in old specimens might have been due to cross-contamination between the reference serum samples as well, but the observed restriction of positive signals to initial samples rather than follow-up samples makes this hypothesis less plausible.

## 4. Materials and Methods

### 4.1. Samples

A total of 1163 residual serum samples were included in the study.

The vast majority of 1137 were presumptive negative control samples for the specificity assessment, collected between 2006 and 2021 and stored frozen at –80°C prior to nucleic acid extraction. These 1137 samples predominantly comprised sera from soldiers returning from tropical deployments [27] but also sera from migrants [28]. Although military deployments in areas of endemicity are associated with an elevated risk of getting infected with *Leishmania* spp. [9], visceral leishmaniasis had never been diagnosed in the included soldier [27] and migrant [28] sub-populations. However, because asymptomatic visceral leishmaniasis had also not been definitely excluded in these individuals, the negative control status had to be considered as presumptive.

The 26 positive control samples were taken from 18 patients with confirmed visceral leishmaniasis. All samples were sent for diagnostic purposes indicating the clinical suspicion of visceral leishmaniasis. Serological titers of positive results in in-house immunofluorescence assays for leishmaniasis were available from all those positive control samples. Third, active infection had been confirmed microscopically or by PCR from the patients’ bone marrow at least once. A sequence-based identification on species level (*Leishmania infantum*) was available in one sample from Switzerland as determined in the routine diagnostic laboratory of the Swiss Tropical and Public Health Institute in Basel, Switzerland. These reference materials had been collected between 1995 and 2021 and stored frozen at –80 °C in the sample collections of the Bernhard Nocht Institute for Tropical Medicine Hamburg, i.e., the German National Reference Center for Tropical Pathogens, and of the Swiss Tropical and Public Health Institute in Basel, Switzerland.

No patient-specific data were included in the study following the ethical clearance for this test comparison study, which only allowed the use of anonymized residual sample materials.

### 4.2. Nucleic Acid Extraction

Nucleic acid extraction was performed using the EZ1 Virus Mini Kit v2.0 Kit (Qiagen, Hilden, Germany) on an automated EZ1 Advanced system (Qiagen, Hilden, Germany), as described previously [40], an assay with which acceptable nucleic acid yields from various human sample types can be achieved [40]. Prior to nucleic acid extraction, all serum samples had been stored frozen at –80 °C for time periods ranging from few weeks to a maximum of 26 years.

### 4.3. PCR Screening Assays

Two in-house real-time PCR assays were applied at the Bundeswehr Hospital Hamburg in Hamburg, Germany, the external site at the Bernhard Nocht Institute, and one at the Bundeswehr Central Hospital in Koblenz, Department XXI, Section B, Germany. In Hamburg, the protocols targeting kinetoplast DNA [15] and the small subunit ribosomal RNA (ssrRNA) gene [16] were applied as described with minor modifications on Rotor-Gene Q (Qiagen) real-time PCR cyclers. In detail, the kinetoplast DNA protocol was run in 20 µL volumes comprising of HotStarTaq Mastermix (Qiagen), a final MgCl_2_ concentration of 3.0 mM, 150 nM of each primer, 60 nM of the probe, and 2.0 µL DNA eluate with an initial denaturation at 95°C for 15 min followed by 45 cycles of denaturation for 15 s at 95 °C and of annealing and amplification for 60 s at 55°C. The ssrRNA-gene protocol was run in 20 µL volumes comprising HotStarTaq Mastermix (Qiagen), a final MgCl_2_ concentration of 2.0 mM, 200 nM of each primer, 20 nM of the probe, and 2.0 µL DNA eluate with an initial denaturation at 95 °C for 15 min followed by 40 cycles of denaturation for 15 s at 95 °C and of annealing and amplification for 60 s at 60 °C. Extraction and inhibition control real-time PCR were based upon a protocol targeting a Phocid Herpes Virus (PhHV) sequence as described [41] provided on a plasmid, which was added to the samples prior to nucleic acid extraction. Each PCR run comprised a positive and negative control. While the negative control was PCR grade water, the positive controls consisted of plasmids with target-specific sequence inserts, as shown in Table A1 in Appendix A. The limits-of-detection were 1 target DNA copy/µL for the real-time PCR targeting kinetoplast DNA [15] and 13 copies/µL for the ssrRNA-gene PCR [16] as defined applying dilution series with the positive control plasmids and the software SciencePrimer.com (http://scienceprimer.com/copy-number-calculator-for-realtime-pcr, accessed on 2 June 2021).

An additional in-house real-time PCR assay targeting the glucose-6-phosphate isomerase (*gpi*) gene of *Leishmania* spp. was applied at the Bundeswehr Central Hospital Koblenz in Koblenz, Department XXI, Section B, Germany. In detail, the PCRs were performed in 20 µL volumes using Takyon No Rox Probe MasterMix (Eurogentec, Seraing, Belgium), 300 nM of each primer, 250 nM of the probe, and 2.5 µL of the DNA eluate on a LightCycler 480 platform (Roche, Mannheim, Germany). Primers (forward primer Leish-fw 5′-GTGATGCTTTCGATYGGCTA-3′, reverse primer Leish-bk 5′-GCCAGCATCATCGGCAA-3′), and probe (5′-CACGTGATGGATAACCACTTTGCG-3′) were synthesized by TibMolBiol (Berlin, Germany). The protocol consisted of an initial denaturation step for 3 min at 95 °C, followed by 45 cycles of 5 s at 95 °C, 5 s at 55 °C, and 15 s at 72 °C. Each PCR run comprised a positive and negative control. The preparation of genomic DNA of laboratory-reared *L. major* strain 159/06 was used as the positive control, PCR grade water served as the negative control. The limit-of-detection was at least 7.35 ng genomic DNA as defined applying a dilution series of the positive control DNA. Based on an approximated *L. major* genome size of 32.8 megabases [42], the calculated number of detected genomes is 2 × 10^5^ per reaction. Inhibition control was performed using the DNA Process Control Kit (Roche) as recommended by the manufacturer.

During the implementation of the real-time PCRs used in this study, negative reactions were obtained with DNA extracted from cultured African and American trypanosomes as well as with DNA from other parasites, such as *Plasmodium* spp. or *Toxoplasma* spp., whose DNA may be found in human blood samples, taken from the reference material storage of the Bernhard Nocht Institute for Tropical Medicine Hamburg.

### 4.4. Statistical Analysis

Due to the low number of positive samples, only descriptive assessments were performed to calculate the sensitivity and specificity of the assays. Non-parametric testing, i.e., Kruskal–Wallis testing with a subsequent application of Dunn’s multiple comparisons tests, was used to compare the cycle threshold values as recorded with the different real-time PCR assays. These calculations were performed with the software GraphPad Instat version 3.06 (GraphPad Software Inc., San Diego, California, USA). Significance was accepted at *p* < 0.05.

## 5. Conclusions

The results of the present comparative study of *Leishmania* spp.-specific real-time PCR assays suggest a reliable sensitivity and specificity of the kinetoplast DNA-PCR from serum for the diagnosis of visceral leishmaniasis while ssrRNA gene-PCR and *gpi*-PCR proved considerably less sensitive for this purpose. Based on these results as well as on the results of previous studies, screening for *Leishmania* spp. kinetoplast DNA in serum in the case of serological results suggesting visceral leishmaniasis or in the case of clinical suspicion can be considered. In the case of a positive result, an additional sampling in order to obtain whole blood or invasive collection of bone marrow may be avoidable. In case of a negative result in spite of ongoing suspicion of visceral leishmaniasis, however, the provided data are insufficient to consider a negative serum PCR as a definite exclusion of active disease. In conclusion, serum PCR targeting *Leishmania* spp. kinetoplast DNA is a valuable piece within the diagnostic puzzle that may avoid additional or invasive sampling. Its potential suitability for therapy control assessments requires further assessment. For study purposes in remote endemicity settings, the combined suitability of serum for *Leishmania* spp. serology and PCR is interesting as well; in particular, the storage and transport of blood serum under resource-limited conditions are more feasible compared to whole blood.

## Figures and Tables

**Table 1:** Real-time PCR results obtained with 26 serum samples taken from 18 patients with confirmed visceral leishmaniasis.

Sample-ID	Initial Sample (I.S.)/Follow-Up Sample (F.U.S.) after Therapy	Time in Months Since Initial Sampling	*Leishmania*-Specific Immuno-Fluoresence Titer	Bone Marrow Positive by Microscopy or PCR	Ct Value ^#^ in the Kinetoplast DNA PCR	Ct Value ^#^ in the Small Subunit Ribosomal RNA Gene PCR	Ct Value ^#^ in the Glucose-6-Phosphate Isomerase Gene PCR
HAM-1	I.S.	n.a.	1:640	Yes	27	28	-
HAM-1_2	F.U.S.	3	1:640	In previous assessment	-	-	-
HAM-2	I.S.	n.a.	>1:1280	Yes	33	32	-
HAM-3	I.S.	n.a.	1:320	Yes	30	29	-
HAM-4	I.S.	n.a.	1:320	Yes	39	-	-
HAM-5	I.S.	n.a.	1:640	Yes	29	29	-
HAM-5_2	F.U.S.	22	1:160	In previous assessment	-	-	-
HAM-6	I.S.	n.a.	1:160	Yes	18	23	36
HAM-6_2	F.U.S.	2	1:640	In previous assessment	-	-	-
HAM-7	I.S.	n.a.	>1:1280	Yes	23	24	39
HAM-8	I.S.	n.a.	1:640	Yes	17	24	39
HAM-9	I.S.	n.a.	>1:1280	Yes *	-	-	-
HAM-10	I.S.	n.a.	>1:1280	Yes	29	-	-
HAM-10_2	F.U.S.	6.5	>1:1280	In previous assessment	-	-	-
HAM-11	I.S.	n.a.	>1:1280	Yes	29	-	-
HAM-11_2	F.U.S.	3	1:640	In previous assessment	-	-	-
HAM-12	I.S.	n.a.	1:320	Yes	16	24	37
HAM-12_2	F.U.S.	7	1:80	In previous assessment	-	-	-
HAM-13	I.S.	n.a.	1:320	Yes	20	26	38
HAM-13_2	F.U.S.	2.5	1:80	In previous assessment	-	-	-
HAM-14	I.S.	n.a.	>1:1280	Yes	22	28	-
BAS-1_2°	F.U.S.	5.5	1:1280	In previous assessment	-	-	-
BAS-2_2°	F.U.S.	4	1:640	In previous assessment	-	-	-
BAS-3_2°	F.U.S.	6	1:1280	In previous assessment°	-	-	-
BAS-4	I.S.	n.a.	1:1280	In subsequent assessment	22	28	-
BAS-4_2	F.U.S.	<0.5	1:1280	Yes	10	17	-

HAM = Samples from the diagnostic routine laboratory at the Bernhard Nocht Institute for Tropical Medicine Hamburg. BAS = Samples obtained from the SWISS Tropical and Public Health Institute. n.a. = not applicable. * High Ct value >35 in the bone marrow. Previous identification of *L. infantum* in the bone marrow 6 months ago. Sufficient sample volumes from the initial samples were unavailable; therefore, only follow-up samples could be included in this study. ^#^ High Ct values indicate low copy numbers and vice versa.

## Data Availability

All relevant data are provided in the manuscript. Raw data can be made available on reasonable demand.

## Appendix A

**Table A1:** Sequence inserts of the positive control plasmids for the *Leishmania* spp.-specific in-house real-time screening PCRs targeting kinetoplast DNA and the small subunit ribosomal RNA gene.

Positive control plasmid sequence insert for kinetoplast DNA-PCR protocol (GenBank Accession Code: EU437405.1)
CCACCCGGCCCTATTTTACACCAACCCCCAGTTTCCCGCCTCGGACCCGATTTTTGAC-ATTTTTGGCCAATTTTTGAACGGGATTTCTGCACCCATTTTTCGATTTTCGCAGAACGCC-CCTACCCGGAGGACCAGAAAAG
Positive control plasmid sequence insert for the small subunit ribosomal RNA gene-PCR protocol (GenBank Accession Code: M81430.1)
AAGTGCTTTCCCATCGCAACCTCGGTTCGGTGTGTGGCGCCTTTGAGGGGTTTAGTGCGT-C

## References

[1] Schwarz N.G., Loderstaedt U., Hahn A., Hinz R., Zautner A.E., Eibach D., Fischer M., Hagen R.M., Frickmann H. (2017). Microbiological laboratory diagnostics of neglected zoonotic diseases (NZDs). Acta Trop..

[2] Oliveira J.G.S., Novais F.O., de Oliveira C.I., Junior A.C.D.C., Campos L.F., da Rocha A.V., Boaventura V., Noronha A., Costa J.M., Barral A. (2005). Polymerase chain reaction (PCR) is highly sensitive for diagnosis of mucosal leishmaniasis. Acta Trop..

[3] Deborggraeve S., Boelaert M., Rijal S., De Doncker S., Dujardin J.-C., Herdewijn P., Buscher P. (2008). Diagnostic accuracy of a newLeishmaniaPCR for clinical visceral leishmaniasis in Nepal and its role in diagnosis of disease. Trop. Med. Int. Health.

[4] Evans T.G., Teixeira M.J., McAuliffe I.T., Vasconcelos I., Vasconcelos A.W., Sousa Ade A., Lima J.W., Pearson R.D. (1992). Epi-demiology of visceral leishmaniasis in northeast Brazil. J. Infect. Dis..

[5] Badaro R., Jones T.C., Carvalho E.M., Sampaio D., Reed S.G., Barral A., Teixeira R., Johnson W.D. (1986). New Perspectives on a Subclinical Form of Visceral Leishmaniasis. J. Infect. Dis..

[6] Badaró R., Jones T.C., Lorenço R., Cerf B.J., Sampaio D., Carvalho E.M., Rocha H., Teixeira R., Johnson W.D. (1986). A pro-spective study of visceral leishmaniasis in an endemic area of Brazil. J. Infect. Dis..

[7] Dereure J., Thanh H.D., Lavabre-Bertrand T., Cartron G., Bastides F., Richard-Lenoble D., Dedet J. (2003). Visceral leishmaniasis. Persistence of parasites in lymph nodes after clinical cure. J. Infect..

[8] Russo R., Laguna F., Lopez-Velez R., Medrano F.J., Rosenthal E., Cacopardo B., Nigro L. (2003). Visceral leishmaniasis in those infected with HIV: Clinical aspects and other opportunistic infections. Ann. Trop. Med. Parasitol..

[9] Mody R.M., Lakhal-Naouar I., E Sherwood J., Koles N.L., Shaw D., Bigley D.P., A Co E.-M., Copeland N.K., Jagodzinski L.L., Mukbel R.M. (2019). Asymptomatic Visceral Leishmania infantum Infection in US Soldiers Deployed to Iraq. Clin. Infect. Dis..

[10] Reithinger R., Dujardin J.-C. (2007). Molecular Diagnosis of Leishmaniasis: Current Status and Future Applications. J. Clin. Microbiol..

[11] Mary C., Faraut F., Drogoul M.P., Xeridat B., Schleinitz N., Cuisenier B., Dumon H. (2006). Reference values for Leishmania in-fantum parasitemia in different clinical presentations: Quantitative polymerase chain reaction for therapeutic monitoring and patient follow-up. Am. J. Trop. Med. Hyg..

[12] Sudarshan M., Weirather J.L., Wilson M., Sundar S. (2011). Study of parasite kinetics with antileishmanial drugs using real-time quantitative PCR in Indian visceral leishmaniasis. J. Antimicrob. Chemother..

[13] Pandey K., Pandey B.D., Mallik A.K., Kaneko O., Uemura H., Kanbara H., Yanagi T., Hirayama K. (2010). Diagnosis of visceral leishmaniasis by polymerase chain reaction of DNA extracted from Giemsa’s solution-stained slides. Parasitol. Res..

[14] Cruz I., Millet A., Carrillo E., Chenik M., Salotra P., Verma S., Veland N., Jara M., Adaui V., Castrillón C. (2013). An approach for interlaboratory comparison of conventional and real-time PCR assays for diagnosis of human leishmaniasis. Exp. Parasitol..

[15] Mary C., Faraut F., Lascombe L., Dumon H. (2004). Quantification of Leishmania infantum DNA by a Real-Time PCR Assay with High Sensitivity. J. Clin. Microbiol..

[16] Wortmann G., Sweeney C., Houng H.S., Aronson N., Stiteler J., Jackson J., Ockenhouse C. (2001). Rapid diagnosis of leishmani-asis by fluorogenic polymerase chain reaction. Am. J. Trop. Med. Hyg..

[17] Wortmann G., Hochberg L., Houng H.H., Sweeney C., Zapor M., Aronson N., Weina P., Ockenhouse C.F. (2005). Rapid identi-fication of Leishmania complexes by a real-time PCR assay. Am. J. Trop. Med. Hyg..

[18] Coleman R.E., Hochberg L.P., Swanson K.I., Lee J.S., McAvin J.C., Moulton J.K., Eddington D.O., Groebner J.L., O’Guinn M.L., Putnam J.L. (2009). Impact of Phlebotomine Sand Flies on U.S. Military Operations at Tallil Air Base, Iraq: 4. Detection and Identification ofLeishmaniaParasites in Sand Flies. J. Med Èntomol..

[19] Ceccarelli M., Diotallevi A., Andreoni F., Vitale F., Galluzzi L., Magnani M. (2018). Exploiting genetic polymorphisms in meta-bolic enzymes for rapid screening of Leishmania infantum genotypes. Parasit. Vectors.

[20] Merdekios B., Pareyn M., Tadesse D., Eligo N., Kassa M., Jacobs B.K.M., Leirs H., Van Geertruyden J.-P., Van Griensven J., Caljon G. (2021). Evaluation of conventional and four real-time PCR methods for the detection of Leishmania on field-collected samples in Ethiopia. PLoS Negl. Trop. Dis..

[21] Martín-Sánchez J., Pineda J.A., Morillas-Márquez F., García-García J.A., Acedo C., Macías J. (2004). Detection of Leishmania in-fantum kinetoplast DNA in peripheral blood from asymptomatic individuals at risk for parenterally transmitted infections: Relationship between polymerase chain reaction results and other Leishmania infection markers. Am. J. Trop. Med. Hyg..

[22] Inga R., De Doncker S., Gomez J., Lopez M., Garcia R., Le Ray D., Arevalo J., Dujardin J.-C. (1998). Relation between variation in copy number of ribosomal RNA encoding genes and size of harbouring chromosomes in Leishmania of subgenus Viannia. Mol. Biochem. Parasitol..

[23] Ceccarelli M., Galluzzi L., Migliazzo A., Magnani M. (2014). Detection and characterization of Leishmania (Leishmania) and Leish-mania (Viannia) by SYBR green-based real-time PCR and high resolution melt analysis targeting kinetoplast minicircle DNA. PLoS ONE.

[24] Fissore C., Delaunay P., Ferrua B., Rosenthal E., Del Giudice P., Aufeuvre J.-P., Le Fichoux Y., Marty P. (2004). Convenience of Serum for Visceral Leishmaniasis Diagnosis by PCR. J. Clin. Microbiol..

[25] de Assis T.S.M., Caligiorne R.B., Romero G.A.S., Rabello A. (2009). Detection of Leishmania kDNA in human serum samples for the diagnosis of visceral leishmaniasis. Trans. R. Soc. Trop. Med. Hyg..

[26] Diagnostik und Therapie der viszeralen Leishmaniasis (Kala Azar). https://www.dtg.org/images/Leitlinien_DTG/Leitlinie_viszerale_Leishmaniasis.pdf.

[27] Schawaller M., Wiemer D., Hagen R.M., Frickmann H. (2020). Infectious diseases in German military personnel after predominantly tropical deployments: A retrospective assessment over 13 years. BMJ Mil. Health.

[28] Maaßen W., Wiemer D., Frey C., Kreuzberg C., Tannich E., Hinz R., Wille A., Fritsch A., Hagen R.M., Frickmann H. (2017). Microbiological screenings for infection control in unaccompanied minor refugees: The German Armed Forces Medical Ser-vice’s experience. Mil. Med. Res..

[29] Leslie T., Saleheen S., Sami M., Mayan I., Mahboob N., Fiekert K., Lenglet A., Ord R., Reithinger R. (2006). Visceral leishmania-sis in Afghanistan. CMAJ..

[30] Kaufer A., Ellis J., Stark D., Barratt J. (2017). The evolution of trypanosomatid taxonomy. Parasites Vectors.

[31] Obwaller A.G., Köhsler M., Poeppl W., Herkner H., Mooseder G., Aspöck H., Walochnik J. (2018). Leishmania infections in Aus-trian soldiers returning from military missions abroad: A cross-sectional study. Clin. Microbiol. Infect..

[32] Lima M.S.D.C., Hartkopf A.C.L., Tsujisaki R.A.D.S., Oshiro E.T., Shapiro J.T., Matos M.D.F.C., Dorval M.E.C. (2018). Isolation and molecular characterization of Leishmania infantum in urine from patients with visceral leishmaniasis in Brazil. Acta Trop..

[33] Pessoa-E-Silva R., Mendonça Trajano-Silva L.A., Lopes da Silva M.A., da Cunha Gonçalves-de-Albuquerque S., de Goes T.C., Silva de Morais R.C., Lopes de Melo F., de Paiva-Cavalcanti M. (2016). Evaluation of urine for Leishmania infantum DNA detection by real-time quantitative PCR. J. Microbiol. Methods..

[34] Veland N., Espinosa D., Valencia B.M., Ramos A.P., Calderon F., Arevalo J., Low D.E., Llanos-Cuentas A., Boggild A.K. (2011). Polymerase chain reaction detection of Leishmania kDNA from the urine of Peruvian patients with cutaneous and mucocu-taneous leishmaniasis. Am. J. Trop. Med. Hyg..

[35] Fisa R., Riera C., López-Chejade P., Molina I., Gállego M., Falcó V., Ribera E., Portús M. (2008). Leishmania infantum DNA detec-tion in urine from patients with visceral leishmaniasis and after treatment control. Am. J. Trop. Med. Hyg..

[36] Silva M.A., Medeiros Z., Soares C.R., Silva E.D., Miranda-Filho D.B., Melo F.L. (2014). A comparison of four DNA extraction protocols for the analysis of urine from patients with visceral leishmaniasis. Rev. Soc. Bras. Med. Trop..

[37] Asfaram S., Teshnizi S.H., Fakhar M., Banimostafavi E.S., Soosaraei M. (2018). Is urine a reliable clinical sample for the diagnosis of human visceral leishmaniasis? A systematic review and meta-analysis. Parasitol. Int..

[38] Pappa S., Kontou P., Bagos P., Braliou G. (2021). Urine-Based Molecular Diagnostic Tests for Leishmaniasis Infection in Human and Canine Populations: A Meta-Analysis. Pathogens.

[39] Bossuyt P.M., Reitsma J.B., E Bruns D., A Gatsonis C., Glasziou P.P., Irwig L., Lijmer J.G., Moher D., Rennie D., de Vet H.C.W. (2015). STARD 2015: An updated list of essential items for reporting diagnostic accuracy studies. BMJ.

[40] Frickmann H., Hinz R., Hagen R.M. (2015). Comparison of an automated nucleic acid extraction system with the column-based procedure. Eur. J. Microbiol. Immunol..

[41] Niesters H.G. (2001). Quantitation of Viral Load Using Real-Time Amplification Techniques. Methods.

[42] Ivens A.C., Peacock C.S., Worthey E.A., Murphy L., Aggarwal G., Berriman M., Sisk E., Rajandream M.-A., Adlem E., Aert R. (2005). The Genome of the Kinetoplastid Parasite, Leishmania major. Science.

